# Introgressive hybridization and latitudinal admixture clines in North Atlantic eels

**DOI:** 10.1186/1471-2148-14-61

**Published:** 2014-03-28

**Authors:** Sébastien Wielgoss, Aude Gilabert, Axel Meyer, Thierry Wirth

**Affiliations:** 1 Lehrstuhl für Zoologie und Evolutionsbiologie, Universität Konstanz, 78457 Konstanz, Germany; 2 Institute of Integrative Biology, ETH Zürich, Universitätsstrasse 16, 8092, Zürich, Switzerland; 3 Department of Ecosystem and Public Health, University of Calgary - Faculty of Veterinary Medicine, 3280 Hospital Drive NW, Calgary, Alberta, Canada; 4 Laboratoire de Biologie intégrative des populations, Ecole Pratique des Hautes Etudes, 75005 Paris, France; 5 Muséum National d’Histoire Naturelle, ISYEB, UMR-CNRS 7205, 75005 16 rue Buffon, 75005 Paris, France

**Keywords:** *Anguilla anguilla*, *Anguilla rostrata*, Gene flow, Isolation-by-distance, Simulation, Migration barriers

## Abstract

**Background:**

Hybridization, the interbreeding of diagnosably divergent species, is a major focus in evolutionary studies. Eels, both from North America and Europe migrate through the Atlantic to mate in a vast, overlapping area in the Sargasso Sea. Due to the lack of direct observation, it is unknown how these species remain reproductively isolated. The detection of inter-species hybrids in Iceland suggests on-going gene flow, but few studies to date have addressed the influence of introgression on genetic differentiation in North Atlantic eels.

**Results:**

Here, we show that while mitochondrial lineages remain completely distinct on both sides of the Atlantic, limited hybridization is detectable with nuclear DNA markers. The nuclear hybridization signal peaks in the northern areas and decreases towards the southern range limits on both continents according to Bayesian assignment analyses. By simulating increasing proportions of both F1 hybrids and admixed individuals from the southern to the northern-most locations, we were able to generate highly significant isolation-by-distance patterns in both cases, reminiscent of previously published data for the European eel. Finally, fitting an isolation-with-migration model to our data supports the hypothesis of recent asymmetric introgression and refutes the alternative hypothesis of ancient polymorphism.

**Conclusions:**

Fluctuating degrees of introgressive hybridization between Atlantic eel species are sufficient to explain temporally varying correlations of geographic and genetic distances reported for populations of the European eel.

## Background

Hybridization, the interbreeding of diagnosably divergent species, is a major focus in evolutionary studies [1-4] as it is a key concept for understanding the demographic and evolutionary cohesiveness of natural populations [5,6]. Hybridization has great potential to rapidly introduce variability into a recipient population, if barriers to recombination can be overcome. Cooper [7] put forth strong experimental evidence that sexual recombination speeds up the origin and spread of adaptations in an artificial environment compared to purely clonal strains of *Escherichia coli*, thus supporting the theoretical predictions from the Fisher-Muller model [8,9]. Based on the evidence gathered from empirical data, including invasive species [10] and signatures of massive horizontal gene transfer in a paradigmatic long-term asexual species [11], it might be generalized that in order to quickly adapt, any mechanism of lateral gene transfer or recombination is highly favored in unstable or novel environments. Importantly, the same might apply for populations overlapping after a phase of allopatry, as is assumed to be the case for North Atlantic eels [12].

The two North Atlantic eel species, *Anguilla anguilla* (European eel) and *A. rostrata* (American eel) both have a remarkable catadromous life-cycle that comprises two long-range migrations through the open ocean, a continental growing phase, and a spawning stage in the Sargasso Sea [13]. Despite the key importance of the marine phase [14], most of the scientific investigations focused on the continental phase, and only few data exist on the oceanic parts of the eel’s life-history. To this day, migration routes and exact spawning sites of eels remain largely hypothetical [15-18]. Fifty years ago, to the great surprise of the scientific community, Tucker [19] suggested that the European eel should be considered an evolutionary dead-end of non-spawning individuals. However, with the advent of new molecular markers the genealogical distinctness of the two species was largely clarified (Table 1). DeLigny and Pantelouris [20] and Avise *et al.*[21] argued for the continuation of the two-species-status, originally proposed by Schmidt [16]. This scenario was reinforced when mitochondrial genome sequences became available [22]. Recently, using miniaturized pop-up satellite archival transmitters (PSAT), a small number of eels could be followed during their spawning migration off the European continental shelf towards the Canary and Azores current system. The authors confirmed the eels’ daily vertical migrations between depths of 200–1000 m at a speed of five to 25 km per day [23].

**Table 1:** Summary of the molecular and population genetics literature for European and American eels

**Author(s)**	**Year**	**Marker(s)**	**Sample Sizes (n)**					**Results**
			**Continental**		**Oceanic**			
			**Europe**	**America**	**Iceland**	**Sargasso**	**Azores**	
**Allozymes**								
Fine *et al.*[24]	1964	Transferrins*	44	0	0	0	0	Candidate markers for eel species differentiation: transferrins
Fine *et al.*[25]	1967	Transferrins*	142	104	0	0	0	Heterogeneity among North Atlantic eels (not significant**)
Sick *et al.*[26]	1967	Haemoglobin	848	666	0	0	0	Polymorphism in American eels only, monomorphy in European eels
Pantelouris *et al.*[27]	1970	Transferrins*	40	0	37	0	0	Differentiation among European continental and Icelandic eels (significant**)
Pantelouris *et al.*[28]	1971	Transferrins*	0	63	96	0	0	Differentiation among American continental and Icelandic eels (significant**)
de Ligny & Pantelouris [20]	1973	MDH	300	70	0	0	25	First available diagnostic marker: MDH;
								Differentiation among American and European continental eels (significant**);
								No differentiation among eels from Azores and Europe.
Williams *et al.*[29]	1973	ADH, PHI, SDH, MDH, EST	0	735	0	0	0	Latitudinal clines at three allozyme markers (MDH invariable)
								ADH & SDH clines establish at larval American eel stages
								PHI cline establishes during freshwater residency of American eels
Koehn & Williams [30]	1978	ADH, PHI, SDH	0	n.d.	0	0	0	Latitudinal clines at SDH & PHI loci temporally stable
								ADH cline unstable and allele frequencies vary among years
Comparini & Rodinò [31]	1980	MDH-2	1079	696	0	126	0	Evidence for two eel species at spawning grounds in the Sargasso Sea
Williams *et al.*[32]	1984	MDH-2	n.d.	n.d.	241	0	0	First indication of genetic hybrids in Iceland
Avise *et al.*[12]	1990	MDH-2	0	0	197	0	0	Evidence for an eel hybrid zone: cyto-nuclear disequilibrium in Iceland
Maes & Volckaert [33]	2002	12 loci	304	0	0	0	0	Evidence against panmixia in European eels: IBD (*r* =0.78; *P* = 0.030)
Maes *et al.*[34]	2006	12 loci	840	0	172	0	0	No interannual differentiation in European eels: no IBT (*r* = 0.0050, *P* > 0.05)
**Mitochondrial DNA**								
Avise *et al.*[21]	1986	RFLP	29	109	0	0	0	Strong evidence for two eel species in the North Atlantic
Avise *et al.*[12]	1990	RFLP	17	27	197	0	0	Evidence for an eel hybrid zone: cyto-nuclear disequilibrium in Iceland
Lintas *et al.*[35]	1998	D-loop	55	0	0	0	0	Extensive variability in European eels
Daemen *et al.*[36]	2001	Cytb	253	0	0	0	0	Latitudinal haplotype diversity cline in European eels
**AFLP**								
Albert *et al.*[37]	2006	373 fragments	186	193	748	0	0	Quantification of total fraction of hybrid eels in Iceland (15.5%);
								Latitudinal gradient of hybrid portions in Iceland;
								Evidence for high portion of later generation hybrids (30%);
								Indication of higher survival rates of hybrid eels in Iceland.
Gagnaire *et al.*[38]	2009	373 fragments	186	193	748	0	0	Evidence for selection and non-neutral introgression
**Microsatellites**								
Daemen *et al.*[36]	2001	5 loci	107	0	0	0	0	Low, significant genetic differentiation in European eels (*F*_*ST*_ = 0.040; *P* < 0.050)
Wirth & Bernatchez [39]	2001	7 loci	561	0	50	0	0	Evidence against panmixia in European eels: IBD (*r* = 0.46; *P* < 0.0070);
								Low, significant genetic differentiation in European eels (*F*_*ST*_ = 0.0017; *P* = 0.0014);
								Genetic intermediacy of Icelandic eels among North Atlantic locations.
Wirth & Bernatchez [40]	2003	7 loci	561	402	50	0	0	Evidence for long-term population decline in North Atlantic eels;
								Differentiation among North Atlantic eels (*F*_*ST*_ = 0.018; *P* < 0.0010);
								No evidence against panmixia in American eels: no IBD (*r* = 0.0030; *P* > 0.40).
Mank & Avise [41]	2003	6 loci	44	68	203	0	0	Mild genetic differentiation among North Atlantic eels (*G*_*ST*_ = 0.055; *SE* = 0.0049);
								Genetic intermediacy of Icelandic eels among North Atlantic locations.
Dannewitz *et al.*[42]	2005	6 loci	2566	0	60	0	0	Temporal instability of IBD pattern in European eels;
								Low genetic differentiation in European eels (*F*_*ST*_ = 0.0014; *P* < 0.010);
								Temporal genetic variation exceeds geographic variation.
Maes *et al.*[34]	2006	6 loci	840	0	172	0	0	Evidence for interannual differentiation in Europe: IBT (*r* = 0.18; *P* = 0.043);
								Contradicts allozyme pattern: no IBT, but IBD.
Palm *et al.*[43]	2009	6 loci	1210	0	0	0	0	No genetic differentiation among same-aged silvering eels between a northern and a southern European locality (*F*_*ST*_ = −0.00003; *P* =0.61);
Als *et al.*[44]	2011	21 loci	0	0	0	388	0	No evidence against panmixia in American or European leptocephali
								(*F*_*ST*_ = 0.00019, *P* = 0.4755): no IBD, nor IBT;
								Evidence for inter-species hybridization in the Sargasso Sea
Côté *et al.*[45]	2013	18 loci	0	2142	0	0	0	No evidence against panmixia in the American eel (*F*_*ST*_ = 0.00009; *P* = 0.998);
								Effective population size for American eels: *N*_*E*_ =10 532 (CI95%: 9 312–11 752)

*Reconsidered by Koehn (1972): listed markers agree with Mendelian inheritance and have objectively interpretable banding patterns.

**Statistically re-evaluated by applying χ^2^-statistics with correct degrees of freedom to test for Hardy-Weinberg-Equilibrium.

Atlantic eel stocks have rapidly declined by over 95% compared to the levels prior to 1980 [46]. As a consequence the European eel has been listed as critically endangered on the IUCN red list [47]. This dramatic decrease is attributable to a combination of factors, including habitat destruction, pollution, and over-fishing [48], climate induced changes in the Gulf Stream circulation [49], as well as the recent introduction and spread of *Anguillicola crassus*, an exotic swimbladder nematode that was initially introduced along with infected Japanese eels [50-55]. Disentangling the population structure of both North Atlantic eel species thus has implications on international eel stock management both from conservation and fisheries perspectives*.*

Advanced molecular tools permitted the collection of larger and more sensitive data sets at the population level (for a schematic, more exhaustive review refer to Table 1). However, despite huge sampling efforts these studies have come to different and even contradictory conclusions. Three independent studies reported isolation-by-distance (IBD) in *A. anguilla* which provided evidence against the long-held paradigm of panmixia for this species based on the measurement of very low, but nevertheless highly significant genetic structure within European eels [33,36,39]. Wirth and Bernatchez [39] suggested that IBD patterns are due to a stable temporal delay of spawning migration in eels from Northern habitats, as distances are markedly extended compared to Western and Southern European populations. Alternatively, more than one spawning area might be used by different populations and, also, different currents might carry the leptocephali larvae back from the Sargasso Sea to their parent’s original freshwater habitats in Europe. Thirdly, albeit seemingly less likely, assortative mating among regional groups might be responsible for the observed IBD patterns. Finally, Maes et al. [34] suggested that, given the random factors affecting spawning success in the open ocean, a sweepstake strategy [56] might explain genetically patchy recruitment in sampling locations across Europe [57], leading to a weak but significant isolation-by-time (IBT) signal.

However, most recent studies did not find statistical support for population structure and rejected both IBD and IBT altogether, thus favoring panmixia in both Atlantic eel species [42-45]. In addition, temporal variation among recruits between different arrival waves within the same years significantly exceeded both the geographic and inter-annual genetic differentiation reported above [57-59]. Several points have been raised to explain the discrepancies between early evidence against panmixia and later studies. Palm *et al.*[43] argued that genetic differentiation in the European eel might be largely explained by uncontrolled temporal variation between juvenile glass eel samples. Dannewitz et al. [42] speculated that the use of different cohorts or life stages in the initial studies with strongest support against panmixia could have lead to artifactual isolation-by-distance patterns. Lastly, after evaluating the weak genetic differentiation among North Atlantic eels using basic summary statistics, Mank and Avise [41] concluded that the large overlap in their allelic frequencies is generated by extensive homoplasy associated with a mutation-driven saturation effect. This argument casted doubts on the usefulness of rapidly evolving microsatellite loci for short term evolutionary and hybridization studies in eels.

While there exists strong empirical evidence for near panmixia in both species of North Atlantic eels, not all questions could be successfully addressed. First, it is difficult to see how saturated markers and patchy cohorts within sampling locations could generate statistically significant patterns of IBD over the whole distributional range [39]. Second, the same markers detected the highest signatures of admixture in Icelandic samples [40,41] are consistent with meristic and genetic data that suggested the existence of hybrids in Northern eel habitats in general [13,37,38]. This pattern suggests the existence of a hybrid zone in the Atlantic that could lead to clines of admixture in both species of eel. In fact, this hypothesis has recently been invigorated by a study that relied on an integrated demographic-genetic model and explicitly accounted for the different levels of larval and adult mixing during oceanic migrations [60]. This model showed that even minimal levels of mixing among initially separate sub-populations during both larval dispersal or adult migration are sufficient to entirely erase any genetic differences among them. Building on this finding, the authors offer two explanations for why the geographic differentiation component might have been overestimated in the past: (i) a limited number of temporal recruits analysed, (ii) introgression through inter-species hybridization and non-random dispersal. There is a growing number of studies that show hybridization with molecular data [12,37,44,61], but no study to date has systematically tested this in the North Atlantic eels across their entire range of distribution. Traditionally, many zoologists are reluctant to consider hybridization as an important evolutionary process that generates new species, since the pre- and post-mating barriers to establishing F1 hybrids are often considerable [62-66] (for a review see [67]). However, there is a growing number of examples of reticulate evolution in nature [62,68], particularly from plants and fungi (e.g., [69-73]). A very well known feature of hybrid zones are coincidental changes at several independent characters, resulting in parallel frequency gradients (clines), and such a linkage is measurably exemplified in North Atlantic eel species, *A. anguilla* and *A. rostrata*. Here, cyto-nuclear disequilibrium has been identified in the narrow zone of species overlap in Iceland [12,29,30,74,75], which scales well with a transition zone based on vertebral counts, a nearly diagnostic trait between the two species of eels [12,32]. More specifically, Avise *et al.*[12] estimated the hybrid fraction in Iceland to be 2 to 4% (Table 2). Later, Albert *et al.*[37] evaluated the extent of hybridization and tested for the occurrence of hybrids beyond the first generations, using 376 AFLP markers. A total hybrid fraction of 15.5% was identified, of which 30% were considered to be later generation hybrids.

**Table 2:** Frequencies of American haplotypes in Iceland

**Reference**	***H***	***N***
Avise *et al.*[12]	0.036	438
Kuroki *et al.*[76]	0.060	311
Our study	0.053	300

*H*, Mitochondrial frequency of *A. rostrata* haplotypes in Iceland*; N,* sampling size.

Here, we investigate the extent of population genetic differentiation, and by performing simulations, ask whether IBD patterns might be produced by increasing inter-species hybridization and admixture clines. Such a scenario would also be able to explain the emergence of an IBT signal and fluctuating genetic structure signatures over time. By combining and extending two available data sets [39,40] genotyped at nine microsatellite markers, we investigated eels sampled from the whole North Atlantic distribution area for signals of inter-species hybridization. In a first step, we inferred the admixture proportions for each individual and tested if the most extreme IBD signal detected in European eels [39] can be explained by latitudinal admixture and introgression clines using a simulation approach. Second, we quantified the amount of gene flow that would be necessary to generate the observed correlations, and, finally, we tested if incomplete lineage sorting or recent introgression are most likely to explain the observed lack of monophyly in most nuclear markers observed so far.

## Results

### Summary statistics

Nine microsatellite loci were used to infer intra-specific relationships among North-Atlantic eels. Inter-specific comparison of genetic differentiation for both *F*_*ST*_ and *R*_*ST*_ estimates were highly congruent with one another at 0.0146 (*P* < 0.001) and 0.0147 (*P* < 0.001), respectively. Thus, microsatellite markers used appear to essentially evolve by addition or removal of simple repeats in a step-wise manner. Overall, levels of observed polymorphism were high in North Atlantic eels, ranging from *H*_*o*_ = 0.38 at locus *Aro121* to *H*_*o*_ = 0.90 at locus *Ang101*, with a mean of *H*_*o*_ = 0.78. The same was true for the average numbers of alleles when correcting for sample sizes, ranging from *A*_*R*_ = 10.9 in *Aro054*, to *A*_*R*_ = 17.9 at locus *Ang114*. None of the 36 pairwise tests for linkage disequilibrium among loci was significant after Bonferroni correction [77]. However, Micro-Checker[78] revealed the presence of null alleles for the two newly added loci *Ang075* and *Aro146*. This observation might explain the moderately high *F*_*IS*_ values for most sampling localities (see Additional file 1) and a systematic deviation from HWE for all populations. However, after correction for null alleles using the algorithm FreeNA[79], all but three out of 21 sampling locations were compatible with HWE after Bonferroni correction [77] (see Additional file 2) and in each case where a significant deviation from HWE was detected, only a single locus contributed to the effect.

### Detection of hybrid eels in Iceland

**Figure 1:**
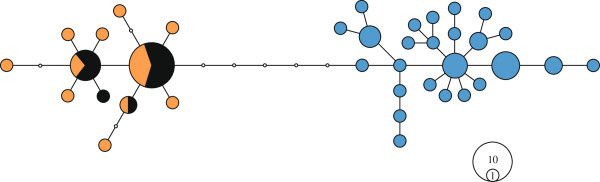
**Minimum-spanning haplotype network based on a partial sequence of *cytb *(276 bp).** The non-overlapping haplotype distribution among continental samples of American (orange, *n* = 15) and European eels (blue, *n* = 34) becomes apparent. A fraction of eels (n = 16) sampled in Iceland carry a typical American haplotype (black), and are thus suspected to be of hybrid origin. The scale on the bottom right-hand side indicates the number of individuals sharing a given haplotype.

**Figure 2:**
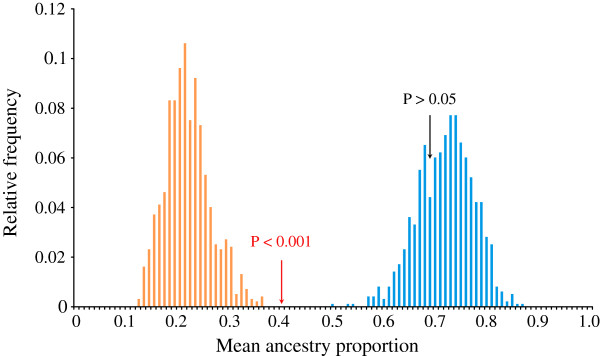
**Suspected hybrids from Iceland are genetically intermediate compared to continental eels.** In analogy to an urn model and in reference to the group of 16 suspected hybrid Icelandic eels, groups of 16 genotypes each were drawn 1000 times from either continental eel population with the mean ancestry proportion calculated for each draw. The permutation test illustrates bimodality, as expected under a two-species model with American eels (orange), and European eels (blue). The mean ancestry proportion of the suspected hybrid eel group is exactly intermediate (red pointer; *Q*_*suspects*_ = 0.40; *P* < 0.001), whereas the mean for Icelandic eels with European haplotypes is not significantly different from the European eel population (black pointer; *Q*_*mean*_ = 0.65; *P* > 0.05).

**Figure 3:**
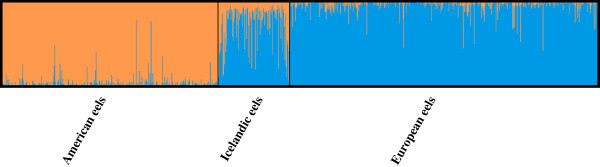
**Bayesian admixture plot highlight on-going hybridization in Iceland.** Nine microsatellite markers were used and the genotypes were analysed using Structure version 2.3.2 [80-82]. Prior geographic information was used for all continental eels comprising American (orange), and European (blue) eels to infer admixture levels in Icelandic eels. Pure species status was accepted for Icelandic individuals when the ancestry proportions *Q* were greater than 0.9. No pure American eel was detected in Iceland.

Based on diagnostic restriction digests of *cytb* fragments, 16 out of 300 Icelandic eels (i.e., 5.3%; Table 2) carried American haplotypes (labelled “suspects”), whereas none of the continental North Atlantic eels showed restriction patterns corresponding to the other species (Figure 1). Thus, as already described, there appears to be a rather clear-cut sorting of mitochondrial lineages with the species boundaries [12,21]. The divergence was less pronounced based on ancestry proportions at nuclear markers, which consistently separated American and European gene pools for the most likely number of populations, *K* = 2. Importantly, the average ancestry proportion of the 16 “suspects” in Iceland (*Q* = 0.40) based on nine microsatellite markers was intermediate (*P* < 0.001) compared to those generated from 1,000 blind draws of 16 random individuals from either species (Figure 2), and thus these suspected mtDNA hybrid individuals most likely represent true F1 hybrids. In addition, the mean ancestry proportion of non-suspect Icelandic eels was not significantly different from the European mean, albeit slightly shifted toward American eels (*Q* = 0.69; *P* = 0.32). When adding prior geographic information on continental stocks, no American expatriate (i.e. *Q* < 0.90 and American mtDNA) was detected in Iceland (Figure 3), but two eels carrying a European mtDNA haplotype were assigned as pure American given their nuclear data.

### Geographic admixture and introgression clines and their impact on IBD patterns

**Figure 4:**
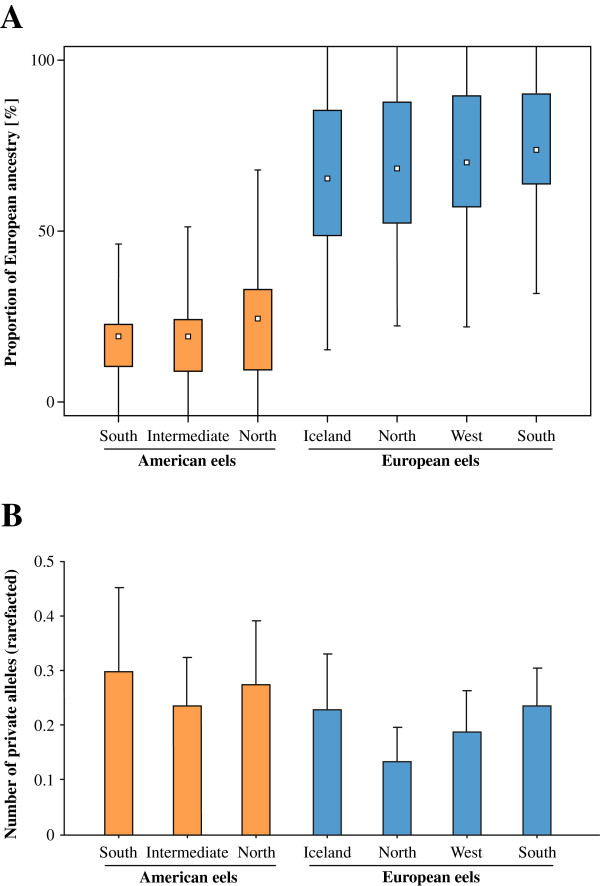
**Geographic admixture clines. (A)** Plots represent clinal geographic change in admixture levels for sampling locations including American (orange), and European (blue) eels. White small boxes represent arithmetic means of admixture proportions, colored boxes delimit the 25%- and 75%-quantiles, respectively, and error bars equal the two-fold standard deviation. Ancestry proportions were inferred from nine microsatellite loci using Structure version 2.3.2 [80-83], the two species were assumed to represent two baseline populations (*K* = 2), without considering the prior information on the species of origin. Values are relative to the European eel samples. Individual eels were partitioned according to distinct geographic entities within continents based on mean surface water temperature categories. **(B)** Number of private alleles in American (orange), and European (blue) eels. Bars represent average numbers after rarefaction for the same geographic partitions as stated above. Error bars correspond to the 95% confidence intervals.

Based on the most highly supported scenario in Structure (*K* = 2; *lnP(D)* = − 48,852.0; see Additional files 3 and 4), the means of ancestry proportions were declining from South to North, whereas the opposite was true for the standard deviations (Figure 4A). As expected, samples from the Northern distribution had the lowest numbers of private alleles on either continent (Figure 4B). Moreover, Iceland had the highest average level in observed heterozygosities (see Additional file 1). Upon correcting for the presence of null alleles using the INA method [79] in FreeNA, similar results were obtained, albeit with different absolute levels. Again, two populations were clearly statistically supported based on the *∆K* statistics for the Structure output (*K* = 2; *lnP(D)* = − 50,775.0; see Additional file 5), and the same geographic cline was apparent over the whole distribution range of the North Atlantic eels (data not shown). The geographic groupings were statistically robust as revealed by pairwise non-parametric tests. Based on the inferred ancestry proportions in Structure, significant differentiation was apparent among four out of the six comparisons among groups within continents. In Europe, differentiation was apparent among Southern and Northern European samples before and after Boxcox transformation (*P* = 0.0190 and 0.00003, respectively), and the same trend was found between the intermediate ranges and the southern locations (*P* = 0.0233). The intermediate ranges were only marginally different from the northern locations (*P* = 0.094). Among American eels, both southern and northern groups were statistically different from the intermediate group after Boxcox transformation (*P* = 0.0005, and *P* = 0.00005, respectively).

**Figure 5:**
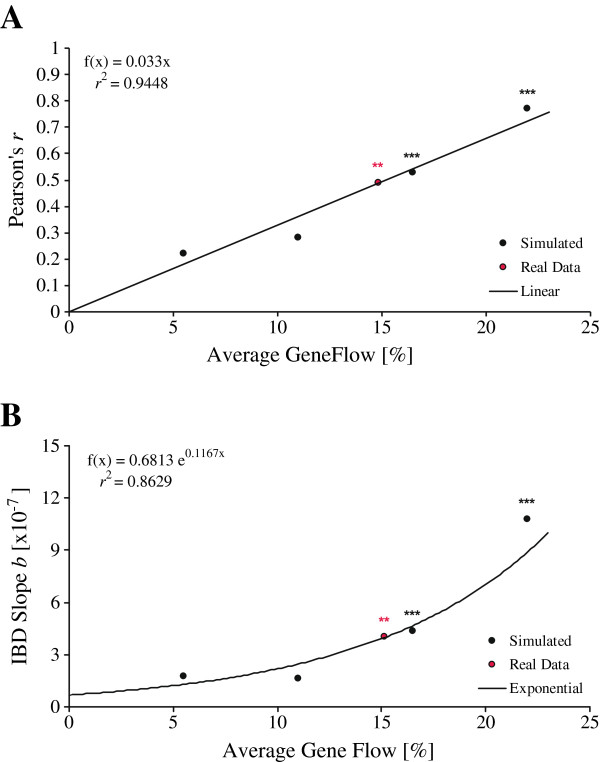
**Inference of gene flow necessary to explain the significant isolation-by-distance patterns in Wirth and Bernatchez **[39]**. (A)** Using best-fit regression based on Pearson’s correlation coefficients *r*; and **(B)** slopes of the trend lines. Gene flow in real data was estimated according to the curve fitting functions.

In line with the geographic admixture cline, we identified members of the southern-most groups of each continent as representing the purest populations: the American population from River St Johns, Florida, and the European population from River Minho, Portugal, respectively. These samples were used as baseline populations for our F1 hybrid simulation approach. Importantly, the simulation of an IBD pattern in the European eel revealed increasing values of both IBD correlation coefficients and significance levels of the Mantel tests (see Additional file 6) when increasing levels of gene flow were applied. Assuming that the real IBD pattern [39] was solely explained by the F1 hybridization cline, an average F1 proportion of ca. 15% among populations with a maximum of 30% in Icelandic eels can explain the IBD signal detected. Whilst a linear regression fit the data best for correlation coefficients *r* (Figure 5A), exponential curve fitting performed slightly better for the slopes of the trend lines *b* (Figure 5B). It might be also worth mentioning that positive IBD correlations largely outnumbered flat or negative correlations (significance not considered here) in the literature; a trend which might have some biological meaning.

However, these numbers exceed by far the literature values reported on clearly evidenced first generation hybrids both at continental ranges [40] as well as in the vicinity of the Sargasso Sea [57]. Thus, our simulation results are not fully consistent with observation of hybrids in nature, and it seems that our assumptions of increasing clines of F1 hybrids alone represents a clear oversimplification.

**Figure 6:**
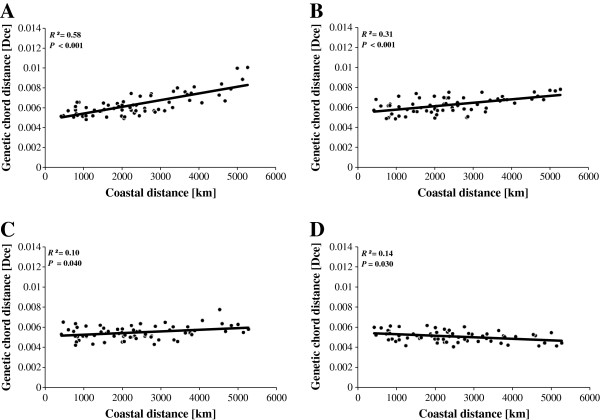
**Influence of admixture clines on isolation-by-distance patterns.** The purest genotypes apparent from inferred admixture values in STRUCTURE were sorted into bins. Eels with extreme *Q*-values were selected as representatives of the two “pure” gene pools (*Q* < 0.1, and *Q* > 0.9 for American and European eels, respectively). The distribution of “admixed” *Q*-values was inferred from remaining intermediate genotypes, and served to inform a random sampling strategy to draw alleles, using multinomial sampling, from the respective “pure” gene pools according to abovementioned “admixed” *Q*-proportions. We augmented the proportion of virtually created “admixed” individuals in a stepwise process by **(A)** 4%; **(B)** 3%; **(C)** 2% and **(D)** 1% per population for a total of 12 virtual populations each. Significance of IBD was tested using the Mantel statistics for correlated genetic data [84]. To test our hypothesis, that IBD patterns can be generated in European eels by increasing levels of gene flow from South to North, the rectangular matrix of pairwise geographical distances from Wirth and Bernatchez [39] was superimposed on the genetic pairwise *D*_*CE*_ chord distances among the 12 virtual populations. Thus, assuming a linear increase of gene flow, we attributed the Southern-Eastern-most location (River Tiber) the lowest, and the Northern-Western-most locality (Iceland) the highest hybridization rate. Intermediate levels were attributed in ascending order along the European coastline.

Hence, in a second, more realistic attempt, we performed a new set of simulations accounting for the observed diversity of admixture values derived from STRUCTURE (see Additional file 3). Following this method we did not produce F1 genotypes with intermediate admixture values, but admixed genotypes satisfying admixture values of near to *Q*_*expected*_ = 0.5 (the actual values of virtual admixed individuals equalled Q_*observed*_ = 0.55 given the real dataset). As a result we were able to recapitulate the same observation and pattern as in analysis 1 (Figure 6).

**Figure 7:**
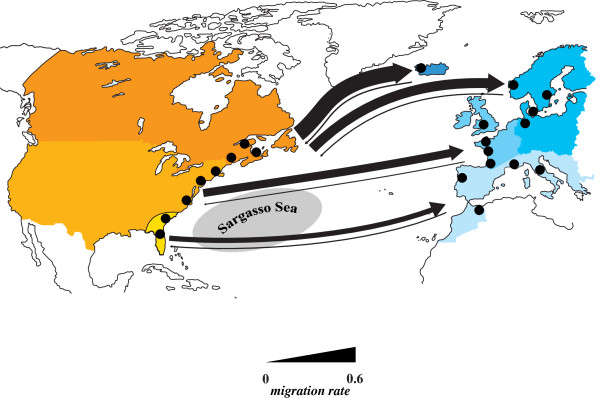
**Schematic representation of the level of gene flow between American (orange coloration; left hand side) and European eels (blue coloration; right hand side).** Arrows depict directionality of the migration parameter *m* from either eel species scaled by the mutation rate (*m* = *M/μ*, with *M* being the migration rate per generation per gene copy, and µ being the mutation rate). Thickness of arrows indicates strength of gene flow, and are to scale with median values from Table 3.

Finally, assuming an IM model as implemented in IMa2, we were able to refute the possibility that incomplete lineage sorting and thus ancient polymorphism skewed our data. We then directly estimated the reciprocal migration rates among the two Atlantic eel species. Chain convergence was achieved in most cases and three independent runs per population pair gave very similar, and thus reproducible results (Table 3). Moreover, posterior probability distributions of the population size and migration rate parameters showed one clear peak with fairly narrow ranges (see Additional file 7). Therefore, although estimated values must be considered with caution, we can reasonably draw some conclusion from these results. Importantly, the data confirm our already observed clinal admixture trend with high migration rates between northern populations and subsequent decreases toward southern pairs (Figure 7; Table 3). The results indicate a ~5 to 10-fold difference in gene flow with a major contribution from American toward European eels, and this for all three continental population pairs (likelihood-ratio tests of migration parameters implemented in IMa2 was not considered here because of the use of an exponential prior for migration rate; see [85,86]). It is worth mentioning that our nine markers have different discriminatory power in detecting IBD as depicted by the variation in locus-specific F_*ST*_ values (see Additional file 8). However, this variation is unlikely to have interfered with our ability to detect hybridization as depicted by the clear results of our various simulation approaches.

**Table 3:** Population size and migration rate parameters

**Parameters**	**Effective population size (4*N***_***i***_***μ*)**		**Migration rate (*M*/*μ *)**		**Population migration rate (2*NM*)**	
**Population pairs**	***Aro***	***Aan***	***Aro *** **to *Aan***	***Aan *** **to *Aro***	***Aro *** **to *Aan***	***Aan *** **to *Aro***
Northern *A. rostrata*/Icelandic *A. anguilla*						
run01	49.3	36.7	0.492	0.035	9.94	1.15
run02	49.1	36.7	0.508	0.037	10.4	1.24
run03	49.0	36.7	0.530	0.031	10.3	1.01
**Median**	**49.1**	**36.7**	**0.508**	**0.035**	**10.3**	**1.15**
Northern *A. rostrata*/Northern *A. anguilla*						
run01	55.9	32.3	0.317	0.052	5.31	1.59
run02	56.5	31.1	0.337	0.038	5.84	1.23
run03	56.5	31.8	0.320	0.027	5.55	0.936
**Median**	**56.5**	**31.8**	**0.320**	**0.038**	**5.55**	**1.23**
Intermediate *A. rostrata*/Western *A. anguilla*						
run01	58.8	37.9	0.276	0.030	5.60	0.996
run02	57.4	38.1	0.258	0.040	5.31	1.15
run03	57.7	37.9	0.260	0.031	5.42	1.33
**Median**	**57.7**	**37.9**	**0.260**	**0.031**	**5.42**	**1.15**
Southern *A. rostrata*/Southern *A. anguilla*						
run01	51.6	50.4	0.173	0.033	4.61	1.2
run02	52.3	51.7	0.167	0.036	4.87	1.27
run03	50.2	51.0	0.162	0.045	4.16	1.25
**Median**	**51.6**	**51.0**	**0.167**	**0.036**	**4.61**	**1.25**

Parameters are estimated from the location of the peaks of the estimated posterior probability densities. Population size and migration rate parameters are scaled by the mutation rate *μ. Aro*, *A. rostrata*; *Aan*, *A.* anguilla; N_i_, the effective size of population i; μ, the mutation rate; M, the migration rate per generation per copy.

## Discussion

The fascinating life history of Atlantic eels that involves a rare catadromous spawning migration, their economic impact and the concomitant international trade has prompted numerous studies in the past 30 years. Therefore it is astonishing that, despite the large efforts made, so many questions still remain unanswered [87]. Our empirical and simulated data suggest that the paradigm of panmixia that was recently wavering due to major population genetics advances might still hold [42-44,61]. Here we propose a scenario consisting of two randomly mating populations with fluctuating and clinal introgression rates; this result alone will affect the large-scale management of both endangered species since first- and later generation hybrids apparently accumulate at Northern habitats.

### Hybridization pattern among North-Atlantic eels

In our attempt to detect hybrids relying on nine microsatellite markers, we came to two major conclusions. First, 16 Icelandic eels sharing an American mtDNA are genetically intermediate (i.e. hybrids) and significantly depart from pure European or American samples (*P* < 0.05). Second, the admixture clines observed on both sides of the Atlantic (Figure 4A) are very unlikely to be obtained by chance alone. Combinational statistics tells us that obtaining a hierarchic rank of 1, 2, 3 from three samples (American eel) has a probability *P* = (1/3!); obtaining a rank of 1, 2, 3, 4 from 4 samples (European eel) has a probability *P* = (1/4!). Therefore by combining these occurrences, we have only one chance in 144 (*P* = 0.007) of obtaining the result shown in Figure 4A. Though, one cannot fully exclude the presence of homoplasy, noise did not erase genetic information as previously assumed [41].

Here we argue that latitudinal clines of admixed individuals between European and American eels might be sufficient to explain IBD patterns observed in the European eel using neutral microsatellite markers [39]. The latitudinal hybrid clines are most likely due to a very recent onset of gene flow after a secondary overlap and could reflect superior hybrid fitness in the northern parts of the Atlantic. The following line of argument supports this inference.

First, we confirm that Icelandic samples display high admixture proportions when compared to the continental samples. Hybrid frequencies in Iceland are high and the proportion of inferred American haplotypes is in good agreement with reported cyto-nuclear disequilibria [12,37,75,88], which is a hallmark of hybrid zones [1,2,74]. Second, while mitochondrial lineages in eels remain 100% distinct on both sides of the Atlantic, the hybridization signal expands further to continental stocks in the nuclear genes, with decreasing latitudinal allelic richness and admixture proportions. Thus, this diffusion most likely depicts recent on-going gene flow introducing new alleles into each continental nuclear gene pool by back-crossing in the absence of maternal lineage mixing. Third, when simulating declining proportions of both F1 hybrids or more realistic fractions of admixed individuals from North to South, a stepwise decrease of approximately 5% of foreign alleles per 1,000 km of coastline would explain the IBD signal reported in Wirth and Bernatchez [39], whereas absent or low gene flow will fail to do so. Fourth, an isolation-with-migration model fits the data better then the alternative hypothesis of clinal ancestral polymorphism due to incomplete lineage sorting. The latter analysis also provides strong evidence for directional gene flow from American toward European eels, which is in good agreement with the absence of isolation-by-distance in the American eel [40,45].

Importantly, previous studies suggest that actual F1 and later generation hybrids are both rare on the continents [37] as well as in the vicinity of the spawning grounds in the Sargasso Sea [44]. Thus, the portions of F1 hybrids needed to explain our finding are not fully consistent with observations in nature. However, assuming a more realistic scenario, we could show that increasing latitudinal introgression clines impacted IBD in an identical way as using actual F1 hybrid clines. This can be explained by the fact that the distribution of *Q*-values of admixed individuals averages a value very close to the one apparent in F1 hybrids (*Q* = 0.55 versus 0.50, respectively).

Given semi-permeable barriers to gene flow (such as is apparent from our results) neutral markers that show intermediate linkage disequilibria with loci involved in reproductive isolation exhibit reduced gene flow compared to other neutral but unlinked markers. Such markers with reduced effective migration can display clining gene flow, as evidenced in tropical eel species which showed both admixture and introgression clines to produce an IBD pattern [89]. While we did not explicitly distinguish between these two different clines in our simulations, we put forth strong evidence that gene flow among Atlantic eel species influence measures of population structure such as IBD.

### Reunifying IBD and IBT signals

In light of our results, we are now in the position to consolidate some of the seemingly contradictory results of recent population genetics studies. Catchment areas for the earliest larval stages (group 0) are clearly overlapping among Atlantic eels ([31,44,90]; but see [91]). In contrast, the number of hybrids sampled in the Sargasso Sea was very low, and centred in the spawning areas of the European eel [44]. Thus, there are reasons to believe that actual spawning areas are partially “allopatric”, possibly due to strong oceanic fronts that separate spawning eels within distinct areas [92]. Interestingly, Albert *et al.*[37] showed that the proportion of hybrids reaching Icelandic waters peaked in 1999 and 2000, while it decreased from 2000 to 2003. This trend could explain the strong patterns of isolation-by-distance in two independent studies that relied on samples from 1997 to 1999 [33,39]; while later studies did not find support for population structure at all [42-44,57-59]. The former could then be expected to lead to the clear pattern of isolation-by-time in genotype data that spans the temporal time-frame in question [34]. Thus, as a working hypothesis, sudden bursts of hybridization and hybrid arrivals could explain temporally unstable patterns of IBD, with a clear signal of IBT. Accordingly, the fluctuations in hybrid recruit proportions would reflect changes in the degree and timing of overlap of spawning grounds in an unstable oceanic environment [62]. Since only a very small percentage (0.8%) of hatched eel larvae is expected to reach suitable habitats within two years of dispersal [93], the lower the overlap of each species spawning grounds, the lower and less significant the correlation coefficients of the IBD signal on either continent would be, such as found in [42], or [43]. A recent modelling approach has highlighted that even minimal mixing among any existing sub-populations in the spawning grounds would entirely erase detectable signatures of genetic divergence in arriving eel recruits at the continents [60]. Thus, among the alternative explanations they offer, we assume that admixture and introgression clines can completely explain our findings.

### Evidence for cyto-nuclear incompatabilites and asymmetric gene flow

Even though Iceland is at the intersect of the North Atlantic eel distributions, Icelandic eels show a clear affinity towards the European eel gene pool: the suspect individuals carrying American haplotypes in Iceland are hybrids, and no pure American migrants were recovered there. This observation is in accordance with the results collected by Albert *et al.*[37] who could not identify pure American eels in Iceland based on a much larger sample size and denser geographic coverage. Recently, Frankowski *et al.*[94] reported the regional sampling of eels with American mitochondria in Europe. However, these eels were sampled from both aquacultures and frequently stocked German rivers. In the absence of nuclear genetic evidence, these eels were most likely introduced American eels, which escaped into natural habitats, reminiscent of the situation in Japan, where imported foreign eels are frequently detected in rivers [95]. If anything, we would assume that stocking activities would rather erase our fine signal of clinal gene flow.

While American and European eels are clearly separated according to mitochondrial data, interbreeding signatures gradually increased toward Northern habitats in our data. This cyto-nuclear disequilibrium had already been noticed earlier by Avise *et al.*[12]. Importantly, using an RNA-seq approach, Gagnaire and colleagues [61] delivered a very intriguing molecular mechanism that could explain this finding, and has implications for asymmetric inter-species hybridization. Two components of the ATP synthase complex, one encoded in the mitochondrium (*mt-atp6*), the other in the nucleus (*atp5c1*), show significant signs of diversifying selection between the two Atlantic eel species possibly due to co-adaptation in at least one of the species. Thus, conflicts between certain cyto-nuclear combinations could render energy metabolism dysfunctional, diminishing the number of possible hybrid combinations as a result. Interestingly, the authors deliver strong evidence for asymmetric gene flow in several nuclear genes, most of which are directed into the European gene pool. This observation is matching our data based on neutral microsatellite markers.

### Dispersal time and development

The most obvious difference between the two North Atlantic eel species is their divergent larval dispersal strategy, coupled with differences in the onset of metamorphosis [46,96,97]. Data for the timing of metamorphosis and recruitment are based on otolith daily increments [98]. However, these calculations provided contrasting results between research groups, and, even more worrying, “back-calculated” larval migration times did not match the field observations [91]. These inconsistencies suggest that daily increments are not suitable for calculating the timing of dispersal, but rather that they provide a descent proxy for the timing of metamorphosis of leptocephali into glass eels, which takes place on the verge of open ocean and continental shelves [13]. Kettle and Haines [93] predicted a minimum of two years for larvae to successfully cross the Atlantic solely by passive drift, which was concordant with the early estimates based on larval growth rates by Schmidt [99]. This would suggest a three- to six-fold longer migration time for the European eel compared to its American congener (*A. rostrata*), which arrives within some 7–12 months ([76,96,98,100-102]; but see [103]). That fact might explain why hybrid eels, are also most abundant at intermediate ranges (with on average intermediate arrival times at the continental shelf; [76]).

### Hybrid fitness and ecological peculiarities in Iceland

In light of the frequent detection of hybrids in Iceland, we speculate on the possibility that Nordic habitats, especially Iceland and Greenland [104] might depict ecotone habitats compared to the much warmer continents. Freshwater habitats in these areas were definitely uninhabitable during extensive glaciation events in the Pleistocene and must have been colonized afterward (i.e., not earlier than 10,000 years ago). It is suggested that environmental peculiarities characterize eel habitats in Iceland, as freshwater temperatures are typically much lower there as compared to most potential continental habitats [37,76]. This anomaly is directly reflected in Icelandic glass eel’s otolith microstructure and microchemistry, which lack both the usual sharp decrease in Sr:Ca ratios and elevated increment accumulations [76]. In addition, it is worth noting that the diffuse otolith increment zone after metamorphosis has never been observed in any other eel species outside Iceland. Thus, environmental opportunity might favour F1 hybrids in Iceland and other Nordic habitats.

In support of this argument, Albert *et al.*[37] quantified the hybrid proportions in both recruiting and resident eel stages over several years. A total of 70% of putative hybrids fell into the first generation category, whereas 30% belonged to later generation hybrids. They observed an approximately two-fold increase in hybrid proportions from the recruiting glass eel to the resident yellow eel stages. These results suggest a higher hybrid survival upon residency. The presence of second and later generation hybrids indeed demonstrates that hybrids transmitted their genes to the next generations and this would explain why the admixture extends further South on both continents.

### Final remarks

The calculations presented here are based on the strongest IBD detected and therefore represent the highest gene flow estimates that could be extrapolated from the genetic data. The age-dependent fitness differences [37] were not taken into account here. Therefore, the hybridization rate is also somewhat biased, given that the northern *A. anguilla* samples in the analysed data set are collected from yellow eels fin clips and not from glass eels. However, as has been mentioned elsewhere [60], temporal inter-cohort variation is not expected to lead to a clinally structured partition of genetic variation, but rather to unordered differentiation patterns. The rather weak traces of admixture detected by Albert *et al.*[37] in the continental samples only moderately fit with our results. However, it is worth noting that the few American eels with a later generation hybrid signature belonged to the most northern samples (Medomak River and Boston Harbour) and as such follow the cline presented here. Moreover, Als *et al.*[44] only detected one hybrid leptocephalus out of 388 eel larvae in the Sargasso Sea. This very low hybrid prevalence is strikingly different from what is observed at later developmental stages in Iceland [37]. Therefore, selection gradients and landscape genetics must be the main evolutionary forces shaping the latitudinal cline.

Leaving aside the statistical issues (running MCMCs with or without prior), the nature of the genetic markers (co-dominant versus dominant), their relative merits and the quantitative aspects, we now have a couple of clear scenarios and hypotheses to test. The fast developing next-generation sequencing field has already provided us with primer molecular data, that open up the opportunity to enlighten the ominous marine part of the eel’s life cycle (SNPs [61]). Our understanding of the evolutionary dynamics of eel stocks and the selective factors that shape introgressive hybridization in North-Atlantic eels is thus currently going through a quantum leap. Especially if the Northern distribution range can be more systematically and extensively sampled from now on, we will be able to appreciate the actual rate of hybridization among the two Atlantic eel species and follow its fluctuation in space and time. This would also have important practical implications in the monitoring of conservation strategies that have been enacted in Regulation (EC) No 1100/2007 by the European Union [105] in response to the dramatic decline in eel recruitment in the past decades [106]. As a prerequisite for appropriate actions, the fact that admixed individuals and hybrids preferentially tend to accumulate in Northern habitats must be accounted for. For example, bursts of increased inter-species hybridization in certain years might lead to increased numbers of northward-bound recruits, and reduced catchment in more Southern ranges.

## Conclusion

Here, we explicitly focused on the influence of hybridization on genetic differentiation signatures in North Atlantic eels. We relied on the available nuclear microsatellite genotypes and mitochondrial sequence data of 1,263 samples from both continental American and European eels as well as from intermediate Iceland. When simulating continuously increasing proportions of F1 hybrid individuals from the southern to the northern-most locations in Europe, highly significant isolation-by-distance patterns arose, that are reminiscent of previously published data [39]. Therefore, introgressive hybridization alone is sufficient to explain the correlation of geographic and genetic distances reported for the European freshwater eel. Moreover, contrasting signals among nuclear and mitochondrial lineages suggest a recent onset of gene flow, most likely after glacial retreat following the last Ice Age (vicariant scenario [12]). Importantly, our findings are in agreement with previous results on genetic isolation patterns in European eels, either based on geography (IBD [33,39]), or inter-annual genetic composition (IBT [34]). If we can assume that the known overlap of the two species’ spawning grounds [90] is annually changing, the IBD signal should decline in some years (with low overlap), and increase in others. This will then automatically lead to an even higher IBT signal among annual recruitment waves within species. The clear separation of mitochondrial lineages might be explained by cyto-nuclear disequilibria recently detected [61]. Thus, the lack of pure American expatriates in Iceland (see also [37]), could be due to the American eel’s much faster ontogenetic development and metamorphosis which might prevent its settlement in this northern region (“early ripe, early rotten”). All in all, evidence for hybrid survival in Iceland [37] favours the introgression hypothesis followed by subsequent backcrossing. This might not only hold for Iceland but for other Nordic regions as well, and might generate a North-to-South-hybrid gradient in both Atlantic eel species.

## Methods

### Samples and data sets combination

A total of 1263 North Atlantic eels were collected in 1999. The study includes 12 European (n = 561), one Icelandic (n = 300) and eight North American (n = 402) samples (see [39,40]); see Additional file 1 for an overview of all eels samples.

### Microsatellite genotyping and summary statistics

Nearly all North Atlantic eel specimens (n = 1042) have been successfully genotyped using nine microsatellite markers. Original genotypes for seven microsatellite loci [39,40] were supplemented with two additional loci, *Ang075* (Genbank AF237903; Primer sequences, Ang075-F, TATCAGGAACTCGATACGCC, and, Ang075-R, ACGCATCACCAGCCCTTGC), and *Aro146* (AF237904; Aro146-F, CAGTTATCCATCTACAGGTG, and, Aro146-R, GAAATAAGAGAATGAGACTCTG). The same genotyping procedure was applied to the other eel species. The fragment sizes were determined by reference to a size standard using the software Genescan version 2.1 and Genotyper version 2.0 (Applied Biosystems Inc., Foster City, CA). Allelic diversity, genetic variation and deviation from Hardy-Weinberg Equilibrium (HWE) were calculated with Genepop on the web [107] and Genetix version 4.05 [108]. All microsatellites were tested for null alleles using Micro-Checker[78]. Allelic diversity and private allelic richness were also inferred after correcting for unequal sampling sizes using HP-rare[109]. Pairwise genetic differentiation was calculated with Arlequin version 3.1 [110] and statistical significance was inferred after 10,000 permutations.

### Mitochondrial DNA sequencing and species identification

All North Atlantic eel samples were screened by PCR-RFLP analysis of a 362 bp segment of the Cytochrome b gene (*cytb*) [111]. This test is based on a diagnostic *HinfI* restriction site, specific to American eels. In order to confirm this quick screening approach, the *cytb* amplicon was directly sequenced in all Iceland individuals with American haplotypes using an ABI 377 automated sequencer (GenBank accession numbers: KJ546041, KJ546042, KJ546043, KJ546044, KJ546045, KJ546046, KJ546047, KJ546048, KJ546049, KJ546050, KJ546051, KJ5x46052, KJ546053, KJ546054, KJ546055, KJ546056). Incorporating known sequence data from Genbank, a haplotype network based on maximum parsimony was constructed in Tcs version 1.20 [112] relying on an alignment of 278 bp (GenBank accession numbers: AB021767, AB021776, AF006714, AF006715, AF006716, AF006717, AF165069, AF172394, AF368238, AF368239, AF368240, AF368241, AF368242, AF368243, AF368244, AF368245, AF368246, AF368247, AF368248, AF368249, AF368250, AF368251, AF368252, AF368253, AF368254, AF485271, AF485272, AF485273, AF485274, AF485275, AF485276, D28775, D84302, EF427617, EF427618, EU223996, EU223997, EU315235, EU315236, EU315237, EU315238, EU315239, EU315240, EU315241, EU315242, EU315243, EU492326, EU492327, M85080). This network was compared to a maximum likelihood tree to correct for ambiguous, multiple connections. In brief, all redundant sequences were removed from the data set, and the best-fit model of sequence evolution (HKY + I + G) was chosen based on the agreement of all Information Criteria (cAIC, AIC2, BIC) used in Modelgenerator version 0.85 [113]. Based on the estimated shape parameter of the γ-distribution, α = 0.02, the proportion of invariable sites, Pinvar = 0.8849, and an expected transition-transversion ratio of 6.28, a phylogenetic maximum-likelihood tree was inferred in PhyML Online version 3.0 [114] and compared to the haplotype network derived by the parsimony approach in Tcs.

### Assessing individual ancestry proportions

Individual ancestry proportions in North Atlantic eel species were estimated using Structure version 2.3.2[80-83] performing 100,000 burn-in steps followed by 1,000,000 MCMC repeats and three iterations to check for Markov chain convergence. The most likely number of populations was assessed using Evanno’s ad-hoc statistic *∆K*[115]. First, we ran an admixture model without priors to infer the individual ancestry proportions and to detect putative clines of admixture over the whole sampling area. Second, an admixture model including prior information on sampling localities was used (except for Icelandic eels which were included without population information), to estimate the admixture proportions more precisely. A threshold level for the posterior probability of *Q* = 0.9 was used, in order to reach maximum assignment efficiency. Due to the low degree of genetic differentiation between the two North Atlantic eel species (Table 1), the efficiency and performance of hybrid identification may have been hampered by the limited number of markers available [116]. Thus, to test if those Icelandic eels with American mitochondrial haplotypes in our sample (n = 16) are actually intermediate rather than pure expatriates, another test, analogous to an urn model, was performed. Groups of 16 individuals were drawn 1000 times at random from either *Anguilla* gene pools, excluding Iceland. Frequencies of average ancestry proportions were plotted, and the average value for the 16 suspected hybrids was compared to each gene pool, and to the average of the remaining Icelandic samples.

### Inference of geographic admixture clines

Support for up to three geographical groupings was apparent from distance-based phenograms [39-41], the European eel samples were clustered roughly according to the 7-year-average of sea surface temperatures (SST) in the North Atlantic Ocean [114], into Northern (Baltic Sea, Elbe, Imsa), Western (Grand-Lieu, Couesnon, Severn) and Southern (Mediterranean Sea, Minho, Adour) groups. Likewise, the American eels were split into three latitudinal groups, Southern (St. Johns River, South Edisto); intermediate (Wye River, Hudson River, Boston Harbour,); and Northern (Prince Edwards Island, Trinité, Medomak River) samples. How biologically meaningful this categorization is was investigated statistically in R 2.10.1, on the basis of genotypes, by extracting each individual’s ancestry proportions from the most highly supported scenario in Structure and assigning them to their respective geographic group. A Boxcox transformation of the data was applied to find the transformation term that maximizes normal distribution of the data, and thus to increase the statistical power of the test. Normality was tested using the Shapiro-Wilk test, and significant differentiation between pairs of continental groups within species was checked for by applying pairwise non-parametric Wilcoxon tests.

### Simulation of hybridization clines and gene flow quantification

We applied a simulation approach to test our hypothesis, that the IBD signal detected in Wirth and Bernatchez [39] for the European eel could have been generated by the steady increase either inter-species F1 hybrids or admixture clines from Southern to Northern Europe. Instead of the real dataset, which comprised eels from 12 sampling locations along the coastlines of Europe, we used a simple mating scheme to generate 12 virtual hybrid populations differing by a constantly increased level of inter-species hybridization. In essence, for assessing the impact of F1 hybrids on IBD signals, we generated the data in five steps: A) We identified the purest sampling localities of each eel species by considering average admixture coefficients as derived from Structure, thereby reducing any confounding effect due to actual admixture. B) We generated 12 synthetic hybrid populations. For the first hybrid population, we did not allow for admixture, and thus generated it by randomly mating 50 “parental” pairs drawn at random from the purest European eel sample (sampling with replacement). C) For the second and each subsequent population, we increased the proportion of American eel parents sampled at random by 1%, that is, we added one additional American eel parent per population for the 50 crossings. D) We followed the same approach for a total of four different gene flow scenarios: linear increase of interspecies hybridization from one population to the next by 1%, 2% 3% and 4%. For each of the four datasets, pairwise genetic *D*_*CE*_ distances [117] were calculated among populations using Phylip version 3.68 [118]. E) Given both significant IBD and that populations follow a cline of admixture from South to North in the real data, we assigned the hypothetical pairwise genetic distance data to the real geographic distances among the genotyped populations. Finally, in order to estimate the gene flow necessary to establish the degree of correlation (and significance) observed in the original dataset [39], both correlation coefficients (*r*) and the slope of the trend line (*b*) were matched against the simulated gene flow levels.

In a second more realistic simulation approach, we created different degrees of admixed genotypes by sampling alleles randomly from the two species gene pools and this in direct proportion given by the actual admixture values apparent from the STRUCTURE output. Following this approach, we obtained virtual admixed genotypes nearly satisfying an average admixture proportion of 50% (expected for F1 hybrids), at *Q* = 0.55. We performed this simulation using an R script relying on packages adegenet [119,120], ade4 [121], ape [122,123], pegas [124] and plyr [125]. The simulation consisted of these principle steps: i) We first sorted the genotype dataset according to *Q*-values inferred using the aforementioned best run in STRUCTURE and set cut-offs for “pure” American (*Q* < 0.1) and “pure” European (*Q* > 0.9) eels. From this we calculated the respective allele frequency spectrum for both species. ii) We used the remaining individuals to estimate the distribution of all admixture proportions, i.e., we assessed the probability density of *Q*, as discretized in bins of equal sizes of 0.05 ranging [0.10; 0.90]. In order to create admixed individuals, we randomly sampled the *Q*-proportions using multinomial sampling from the observed proportions of *Q*-values in each bin. More specifically, for any given admixed genotype with a certain *Q*-value the appropriate proportion of alleles inherited from either *A. rostrata*, or *A. anguilla* were determined for all nine loci using binomial sampling, and then, using multinomial sampling, alleles were randomly drawn from the allele frequency spectrum of each locus in each “pure species” pool. iii) Finally, we assigned linearly increasing fractions of admixed genotypes of 1%, 2%, 3% or 4%, analogous to the simplified simulation using F1 hybrids, and this again for 100 virtual individuals per population with European eels being the focal species receiving introgressed American alleles.

Lastly, we used the Isolation-with-Migration model implemented in the software IMa2 [85] to test the hypothesis that the pattern observed in Wirth and Bernatchez [39] results from varying gene flow rates between European and American populations as opposed to ancestral polymorphism due to incomplete lineage sorting. By means of Markov Chain Monte Carlo simulations, migration rates (*m = M/μ* with *M* being the mutation rate per generation per gene copy and *μ* the mutation rate), effective population sizes (*q* = 4*N*_*i*_*μ* with *N*_*i*_, the effective size of population *i*) and divergence time (*t = Tμ* where *T* is the time since the common ancestry) were estimated, which allowed us to distinguish gene flow from the other two estimated demographic parameters that are dependent on ancestral polymorphism. IMa2 can deal with more than two populations, but it requires a very large number of loci [85]. Thus, we favoured a two-population model and analysed four pairs of population to estimate migration rates between: a) northern *A. rostrata* and Icelandic populations; b) northern populations; c) intermediate populations; and d) southern populations of *A. rostrata* and *A. anguilla*. Data sets included all genetic markers, namely, the nine microsatellite loci, and the *cytb* fragment reduced to the part containing the recognition site, because this 4-base pair stretch was available for the total dataset. Mutation models used for analyses were a stepwise mutation model for microsatellite loci and the HKY model for the *cytb* fragment. We used an exponential prior on migration to explore both low and high migration rates, even with the modest number of microsatellites available. Moreover, this model makes tests of migration more conservative than a uniform prior [85,126]. Preliminary runs were performed to set the upper bounds (or the mean for migration rate) of the prior distribution of each parameter. The upper limit for the prior for the population size and divergence time parameters were set to 500 and 100, respectively (except for the population pair northern *A. rostrata*-Icelandic population for which the prior for *t* was 30) and the mean of the exponential distribution for the migration rate parameter was set to 2. Since none of the tested values gave satisfying swapping rates between successive chains, we did not run multiple Metropolis-coupled chains and instead, conducted runs with burn-in periods of 30 million followed by a chain of more than 200 million steps, and this for three independent runs with different random number seeds. To assure consistency of results across runs and test for convergence of chains, we compared autocorrelations of values over time, as well as parameter values generated for both the first and second halves of each run [126].

### Availability of supporting data

The data sets supporting the results of this article are available in TreeBASE, http://purl.org/phylo/treebase/phylows/study/TB2:S15621, and LabArchives, http://dx.doi.org/10.6070/H4FX77C8.

## Abbreviations

Bp: Base-pair; Bx: Back-cross; cytb: Cytochrome b; DCE: Cavalli-Sforza & Edwards’s genetic distance; F1: First-generation offsprings; FST: Fixation index; HKY: Hasegawa Kishino and Yano’s molecular genetic substitution model; HE: Expected heterozygosity; HO: Observed heterozygosity; HWE: Hardy-Weinberg equilibrium; IBD: Isolation-by-distance; IBT: Isolation-by-time; IM: Isolation-with-migration; IMa2: Isolation-with-migration program version 2; K: Number of populations; ΔK: Evanno’s ad-hoc statistic; μ: Mutation rate; m: Migration rate; MCMC: Markov chain monte Carlo; mtDNA: Mitochondrial DNA; P: Significance level; Q: Admixture coefficient; r: Correlation coefficient; RST: Slatkin’s fixation index; SE: Standard error; SST: Sea surface temperature.

## Competing interests

The authors declare that they have no competing interests.

## Authors’ contributions

SW performed statistical analyses of the data, wrote the R scripts needed for conducting the hybrid cline simulations, and drafted and completed the manuscript. AG performed IMa2 analyses, and edited the manuscript. AM provided lab material and equipment and edited the manuscript. TW collected the eel samples, performed the lab work, analysed the RFLP and microsatellite data and edited the manuscript. All authors read and approved the final manuscript.

## Supplementary Material

Additional file 1 Average genotypic and allelic diversity for North Atlantic eels specified for each sampling location. Click here for file

Additional file 2 **Average genotypic and allelic diversity for North Atlantic, specified for each sampling location after correcting for null alleles using the INA method according to **[79]**.** Click here for file

Additional file 3 **Relative frequency distribution of admixture levels.** According to Structure version 2.3.2 [80-83], overall high levels of admixture in North Atlantic eels become apparent. Ancestry proportions are illustrated separately for (A) European (blue bars); (B) Icelandic (blue bars) and (C) American eels (orange bars). Click here for file

Additional file 4 **Best-fit for the number of populations (*K*) determined with the Evanno’s ad-hoc statistic *∆K *** [115]**.** (A) The likelihood scores for the different K values were obtained using Structure version 2.3.2 [80-83]. (B) Corresponding values for the ad-hoc statistic *∆K.* This data set corresponds to the nine uncorrected North Atlantic eel genotypes (no null-allele treatment). Click here for file

Additional file 5 **Best-fit for the number of populations (*K*) determined with the Evanno’s ad-hoc statistic *∆K ***[115]**.** (A) The likelihood scores for the different K values were obtained using Structure version 2.3.2 [80-83]. (B) Corresponding values for the ad-hoc statistic *∆K.* This data set corresponds to nine corrected North Atlantic eel genotypes (null-allele treatment using the INA method [79]). Click here for file

Additional file 6 **Influence of F1 admixture clines on isolation-by-distance patterns.** The purest North Atlantic eel locations (River Minho, (PT), *n* = 43; St. Johns River, Fl. (US), *n* = 35) served as parental gene pools for the first generation crosses. We augmented the proportion of F1 hybrids in a stepwise process by (A) 4%; (B) 3%; (C) 2% and (D) 1% per population for a total of 12 virtual F1 populations. Significance of IBD was tested using the Mantel statistics for correlated genetic data [84]. To test our hypothesis, that IBD patterns can be generated in European eels by increasing levels of gene flow from South to North, the rectangular matrix of pairwise geographical distances from Wirth and Bernatchez [39] was superimposed on the genetic pairwise *D*_*CE*_ distances among the 12 virtual populations. Thus, assuming a linear increase of gene flow, we attributed the South-Eastern-most location (River Tiber) the lowest, and the North-Western-most locality (Iceland) the highest hybridization rates, respectively. Intermediate levels were attributed in ascending order along the European coastline. Click here for file

Additional file 7 **Posterior probability distributions for the migration parameters estimated by IMa2 during the first run.** (A) Posterior probability estimates for the migration rate (*M*/*μ*). (B) Posterior probability estimates for the population migration rates (2*NM*). Migration parameters correspond to the rate at which European populations receive genes from American populations. NA (Northern), IA (Intermediate) and SA (Southern) *A. rostrata* populations; NE (Northern), WE (Western) and SA (Southern) *A. anguilla* populations; and ICE (Icelandic population). Click here for file

Additional file 8 **Discriminatory power of microsatellite markers to detect IBD patterns as depicted by locus-specific *F***_***ST ***_**values.** Click here for file
