# Supplementary material for: Introgressive hybridization and latitudinal admixture clines in North Atlantic eels
Source: BMC Evol Biol. 2014 Mar 28;14:61. doi: 10.1186/1471-2148-14-61 (PMC3986858; doi:10.1186/1471-2148-14-61)
Supplement: Additional file 1 — Average genotypic and allelic diversity for North Atlantic eels specified for each sampling location. [file 1471-2148-14-61-S1.pdf]

Additional file 1.

Average genotypic and allelic diversity for Atlantic eels specified for each sampling location.

Species	Location	2n	*A*	*SD*	A_R	*SD*	H_E	*SD*	H_O	*SD*	F_{IS}
A. anguilla	River Tiber (IT)	88	16.8	2.82	13.7	1.99	0.896	0.031	0.782	0.193	0.127*
	Lagoon Salse-Leucate (FR)	82	17.7	3.77	14.5	2.74	0.901	0.031	0.771	0.179	0.144*
	River Moulouya (MA)	100	18.2	3.56	13.8	1.82	0.901	0.020	0.786	0.139	0.128*
	River Minho (PT)	98	17.9	3.95	14.0	2.68	0.898	0.024	0.759	0.199	0.155*
	River Adour (FR)	98	19.0	4.18	14.4	2.65	0.903	0.024	0.807	0.129	0.106*
	Lake Grand-Lieu (FR)	94	17.4	3.24	13.9	1.96	0.903	0.030	0.782	0.205	0.134*
	River Couesnon (FR)	100	18.0	3.12	14.1	2.08	0.900	0.029	0.775	0.190	0.139*
	River Severn (GB)	100	17.8	4.24	13.9	2.44	0.908	0.023	0.813	0.155	0.105*
	River Elbe (DE)	100	18.2	4.35	14.1	2.91	0.904	0.027	0.810	0.127	0.104*
	Lake Arresø (DK)	96	17.6	3.75	13.5	2.77	0.880	0.078	0.811	0.211	0.078*
	Lake Vättern (SE)	48	12.8	2.44	12.3	2.23	0.820	0.166	0.723	0.203	0.118*
	River Imsa (NO)	74	15.6	3.78	13.4	2.80	0.882	0.075	0.775	0.177	0.121*
	River Oelfusa (IS)	250	23.6	5.13	14.2	2.05	0.904	0.026	0.831	0.155	0.081*
A. rostrata	River Petite Trinité, QC (CA)	96	17.8	5.78	13.6	4.30	0.870	0.108	0.719	0.208	0.174*
	Prince Edwards Island, PE (CA)	86	18.0	5.48	14.8	4.08	0.887	0.104	0.752	0.188	0.152*
	River Medomak, ME (US)	92	17.3	5.32	14.2	4.08	0.887	0.096	0.777	0.208	0.124*
	Boston Harbor, MA (US)	98	18.3	6.12	14.5	4.56	0.881	0.125	0.766	0.217	0.131*
	River Hudson, NJ (US)	92	17.6	5.13	14.2	4.14	0.860	0.175	0.772	0.164	0.102*
	River Wye, MD (US)	94	18.0	5.10	14.2	4.18	0.846	0.203	0.762	0.223	0.099*
	River South Edisto, SC (US)	98	18.0	6.24	14.0	4.15	0.879	0.087	0.761	0.184	0.134*
	River St. Johns, FL (US)	98	19.4	6.62	14.6	4.54	0.880	0.119	0.793	0.173	0.099*

significant deviation from HWE after Bonferroni correction; 2n, gene number;* A_R, average number of alleles after rarefaction*; SD, Standard deviation; A*, average number alleles; H_E, unbiased average of expected heterozygosity; H_O, average observed heterozygosity; F_{IS}, inbreeding coefficient. Sampling locations: IT, Italy; FR, France; MA, Morocco; PT, Portugal; GB, Great Britain; DK, Denmark; SE, Sweden; NO, Norway; IS, Iceland; CA, Canada; QC, Province of Quebec; PE, Province of Prince Edwards Island; US, Unites States of America; ME, State of Maine; MA, State of Maine; NJ, State of New Jersey; MD, State of Maryland; SC, State of South Carolina; FL, State of Florida.
