# Supplementary material for: Introgressive hybridization and latitudinal admixture clines in North Atlantic eels
Source: BMC Evol Biol. 2014 Mar 28;14:61. doi: 10.1186/1471-2148-14-61 (PMC3986858; doi:10.1186/1471-2148-14-61)
Supplement: Additional file 2 — Average genotypic and allelic diversity for North Atlantic, specified for each sampling location after correcting for null alleles using the INA method according to [79]. [file 1471-2148-14-61-S2.pdf]

Additional file 2.

Average genotypic and allelic diversity for North-Atlantic, specified for each sampling location after correcting for null alleles using the INA method according to Chapuis and Estoup (2007).

Species	Location	2n	*A*	*SD*	A_R	*SD*	H_E	*SD*	H_O	*SD*	F_{IS}
A. anguilla	River Tiber (IT)	88	16.8	2.82	13.7	1.99	0.897	0.032	0.907	0.035	-0.011
	Lagoon Salse-Leucate (FR)	82	17.7	3.77	14.5	2.74	0.900	0.028	0.902	0.040	-0.003
	River Moulouya (MA)	100	18.2	3.56	13.8	1.82	0.903	0.019	0.904	0.034	-0.001
	River Minho (PT)	98	17.9	3.95	14.0	2.68	0.895	0.027	0.909	0.052	-0.016
	River Adour (FR)	98	19.0	4.18	14.4	2.65	0.905	0.022	0.900	0.039	0.006
	Lake Grand-Lieu (FR)	94	17.4	3.24	13.9	1.96	0.904	0.031	0.913	0.042	-0.009
	River Couesnon (FR)	100	18.0	3.12	14.1	2.08	0.897	0.029	0.909	0.023	-0.014
	River Severn (GB)	100	17.8	4.24	13.9	2.44	0.906	0.024	0.924	0.022	-0.021
	River Elbe (DE)	100	18.2	4.35	14.1	2.91	0.904	0.028	0.904	0.055	<0.001
	Lake Arresø (DK)	96	17.6	3.75	13.5	2.77	0.894	0.048	0.903	0.077	-0.010*
	Lake Vättern (SE)	48	12.8	2.44	12.3	2.23	0.853	0.088	0.847	0.112	0.007
	River Imsa (NO)	74	15.6	3.78	13.4	2.80	0.885	0.058	0.886	0.069	-0.002
	River Oelfusa (IS)	250	23.6	5.13	14.2	2.05	0.907	0.020	0.917	0.023	-0.012
A. rostrata	River Petite Trinité, QC (CA)	96	17.8	5.78	13.6	4.30	0.890	0.070	0.875	0.091	0.017*
	Prince Edwards Island, PE (CA)	86	18.0	5.48	14.8	4.08	0.902	0.074	0.912	0.077	-0.011
	River Medomak, ME (US)	92	17.3	5.32	14.2	4.08	0.882	0.076	0.855	0.126	0.030*
	Boston Harbor, MA (US)	98	18.3	6.12	14.5	4.56	0.896	0.086	0.900	0.081	<0.001
	River Hudson, NJ (US)	92	17.6	5.13	14.2	4.14	0.867	0.159	0.855	0.161	0.014
	River Wye, MD (US)	94	18.0	5.10	14.2	4.18	0.863	0.164	0.858	0.162	0.005
	River South Edisto, SC (US)	98	18.0	6.24	14.0	4.15	0.890	0.062	0.907	0.073	-0.019
	River St. Johns, FL (US)	98	19.4	6.62	14.6	4.54	0.893	0.093	0.891	0.098	0.002

significant deviation from HWE after Bonferroni correction; 2n, gene number;* A_R, average number of alleles after rarefaction*; SD, Standard deviation; A*, average number alleles; H_E, unbiased average of expected heterozygosity; H_O, average observed heterozygosity; F_{IS}, inbreeding coefficient. Sampling locations: IT, Italy; FR, France; MA, Morocco; PT, Portugal; GB, Great Britain; DK, Denmark; SE, Sweden; NO, Norway; IS, Iceland; CA, Canada; QC, Province of Quebec; PE, Province of Prince Edwards Island; US, Unites States of America; ME, State of Maine; MA, State of Maine; NJ, State of New Jersey; MD, State of Maryland; SC, State of South Carolina; FL, State of Florida.
