# Supplementary figures and images for: Introgressive hybridization and latitudinal admixture clines in North Atlantic eels
Source: BMC Evol Biol. 2014 Mar 28;14:61. doi: 10.1186/1471-2148-14-61 (PMC3986858; doi:10.1186/1471-2148-14-61)

**Additional file 3.**

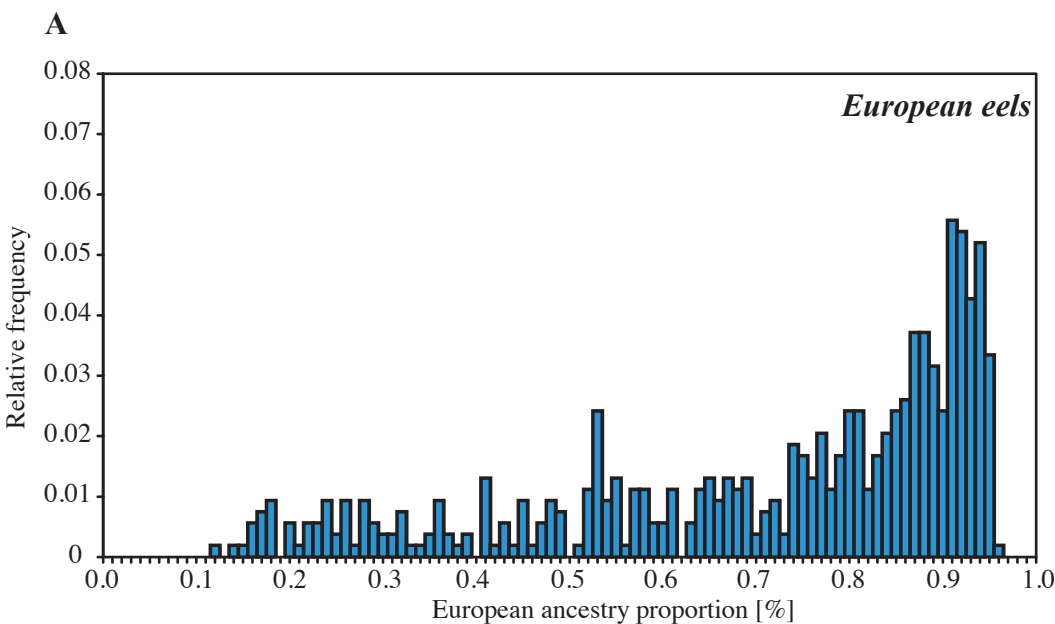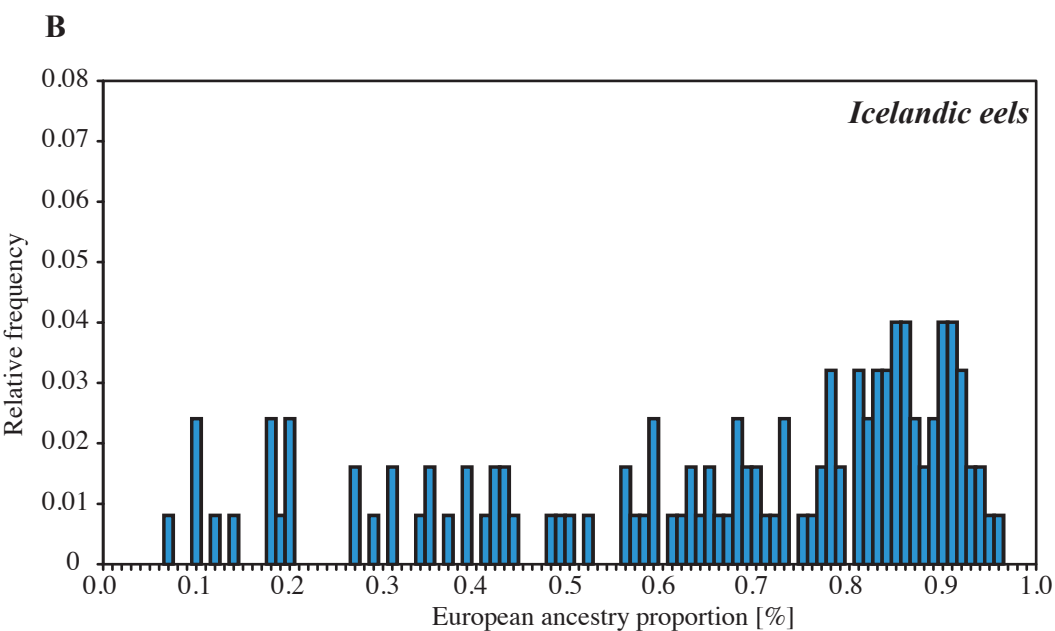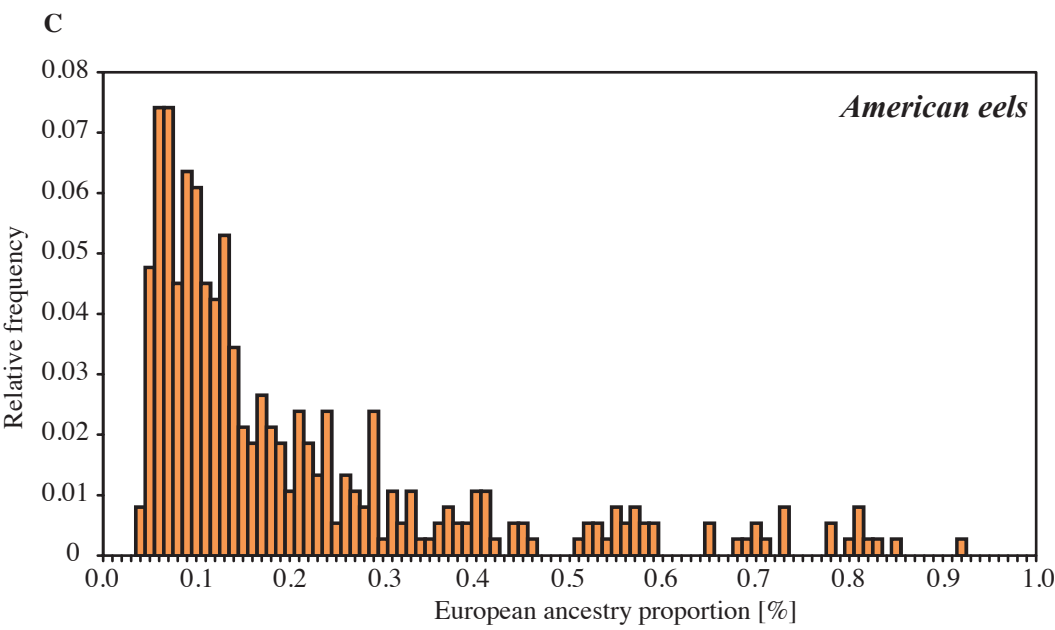

Supplement: Additional file 3 — Relative frequency distribution of admixture levels. According to Structure version 2.3.2 [80-83], overall high levels of admixture in North Atlantic eels become apparent. Ancestry proportions are illustrated separately for (A) European (blue bars); (B) Icelandic (blue bars) and (C) American eels (orange bars). [file 1471-2148-14-61-S3.pdf]

**A**

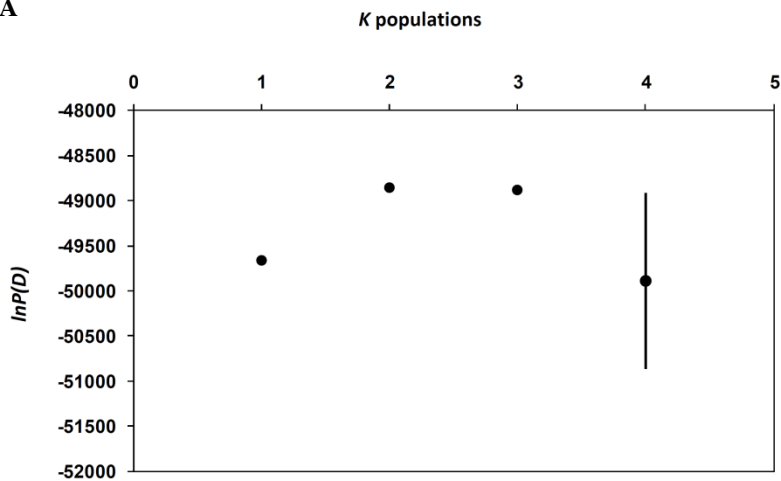

**B**

Evanno's  $\Delta K$

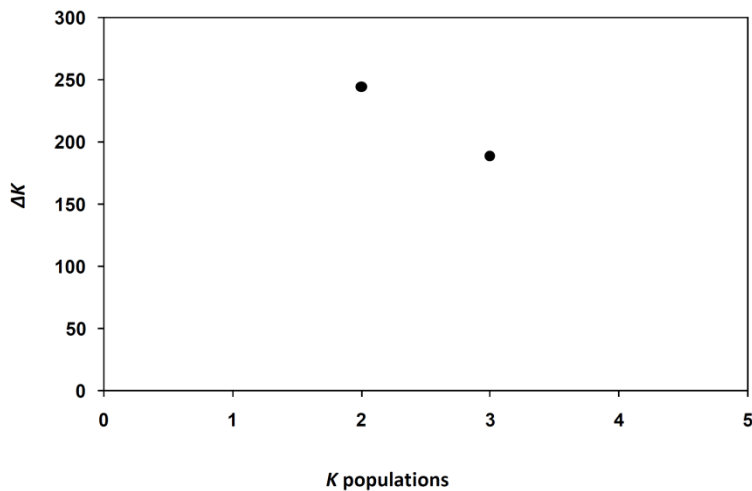

Supplement: Additional file 4 — Best-fit for the number of populations (K) determined with the Evanno’s ad-hoc statistic ∆K [115]. (A) The likelihood scores for the different K values were obtained using Structure version 2.3.2 [80-83]. (B) Corresponding values for the ad-hoc statistic ∆K. This data set corresponds to the nine uncorrected North Atlantic eel genotypes (no null-allele treatment). [file 1471-2148-14-61-S4.pdf]

## Additional file 5.

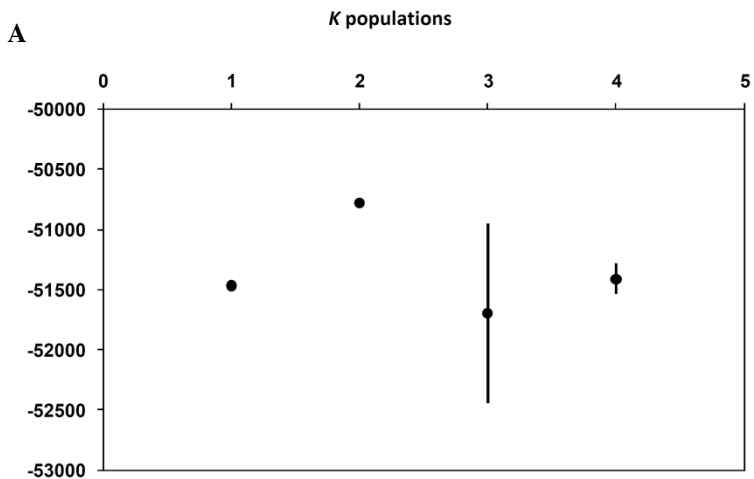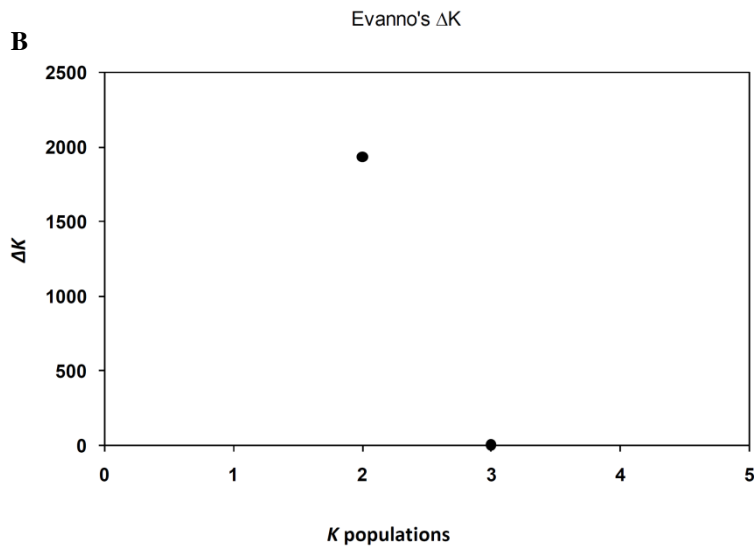

Supplement: Additional file 5 — Best-fit for the number of populations (K) determined with the Evanno’s ad-hoc statistic ∆K [115]. (A) The likelihood scores for the different K values were obtained using Structure version 2.3.2 [80-83]. (B) Corresponding values for the ad-hoc statistic ∆K. This data set corresponds to nine corrected North Atlantic eel genotypes (null-allele treatment using the INA method [79]). [file 1471-2148-14-61-S5.pdf]

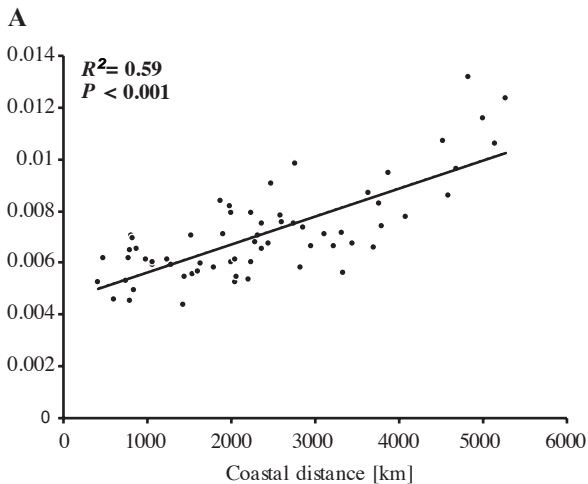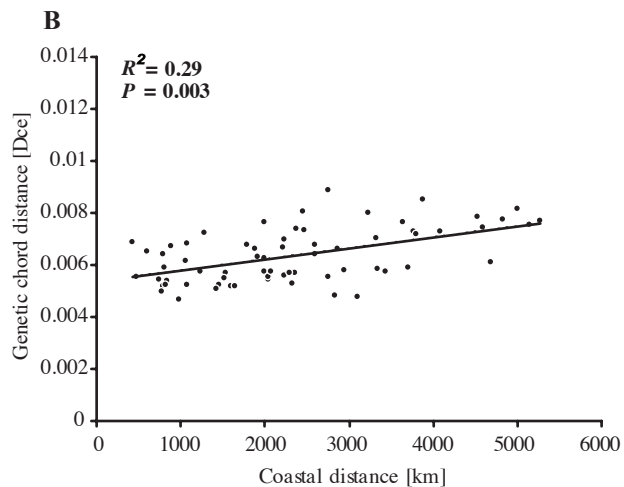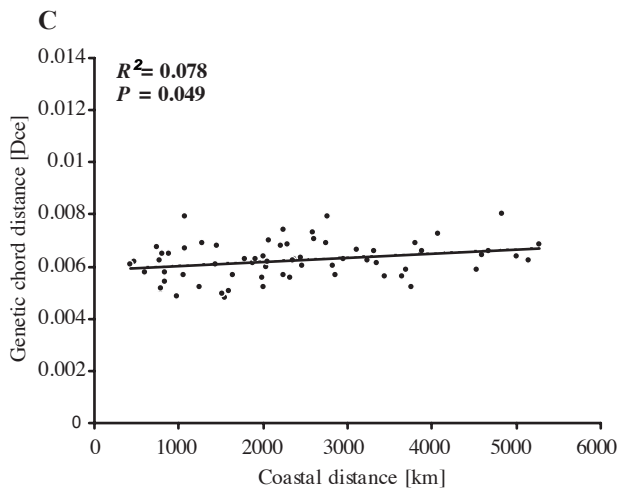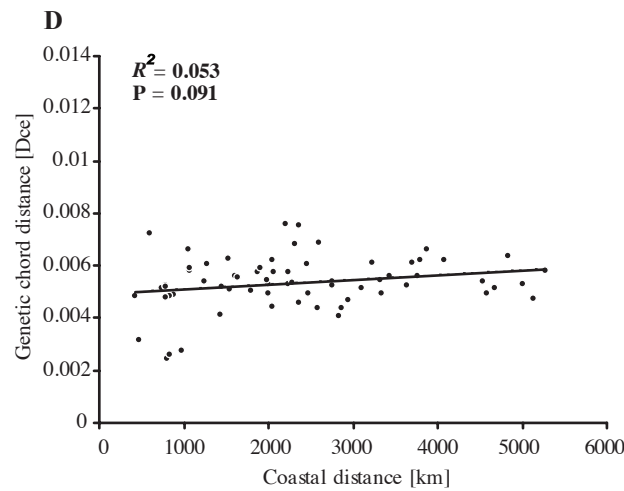

Supplement: Additional file 6 — Influence of F1 admixture clines on isolation-by-distance patterns. The purest North Atlantic eel locations (River Minho, (PT), n = 43; St. Johns River, Fl. (US), n = 35) served as parental gene pools for the first generation crosses. We augmented the proportion of F1 hybrids in a stepwise process by (A) 4%; (B) 3%; (C) 2% and (D) 1% per population for a total of 12 virtual F1 populations. Significance of IBD was tested using the Mantel statistics for correlated genetic data [84]. To test our hypothesis, that IBD patterns can be generated in European eels by increasing levels of gene flow from South to North, the rectangular matrix of pairwise geographical distances from Wirth and Bernatchez [39] was superimposed on the genetic pairwise DCE distances among the 12 virtual populations. Thus, assuming a linear increase of gene flow, we attributed the South-Eastern-most location (River Tiber) the lowest, and the North-Western-most locality (Iceland) the highest hybridization rates, respectively. Intermediate levels were attributed in ascending order along the European coastline. [file 1471-2148-14-61-S6.pdf]

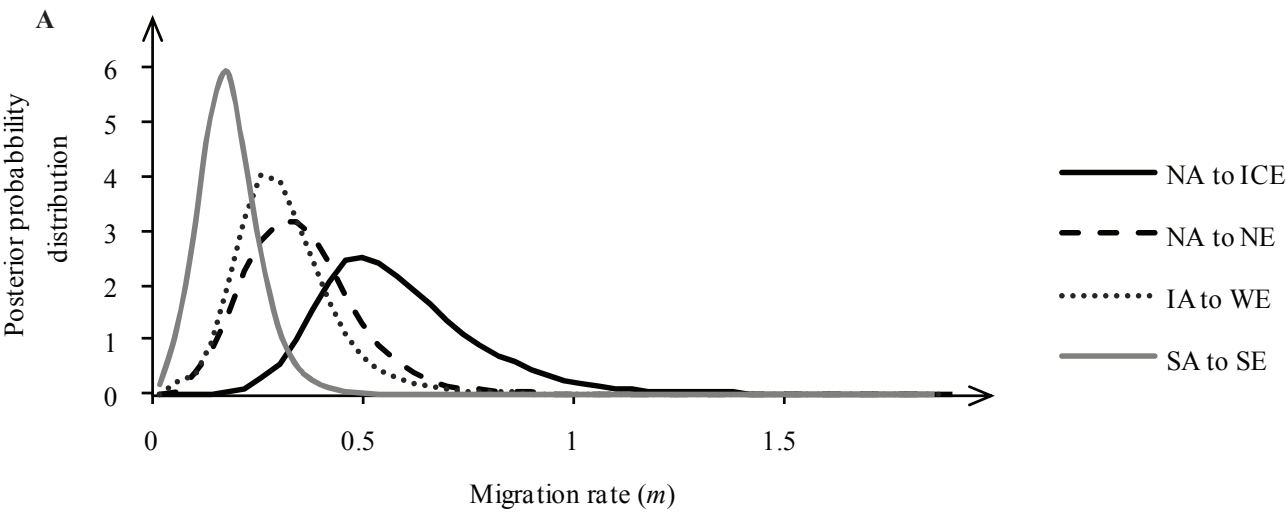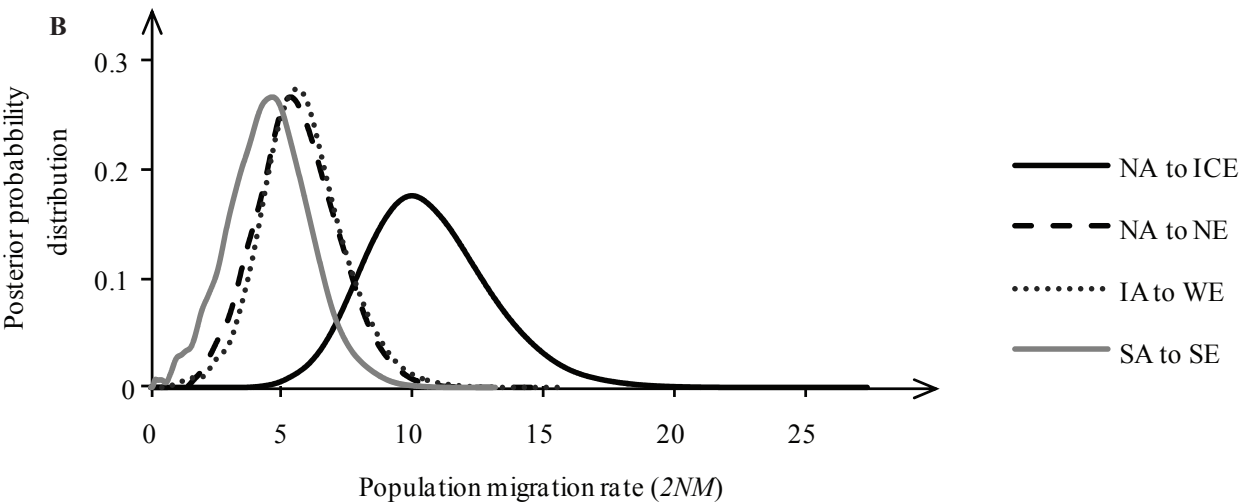

Supplement: Additional file 7 — Posterior probability distributions for the migration parameters estimated by IMa2 during the first run. (A) Posterior probability estimates for the migration rate (M/μ). (B) Posterior probability estimates for the population migration rates (2NM). Migration parameters correspond to the rate at which European populations receive genes from American populations. NA (Northern), IA (Intermediate) and SA (Southern) A. rostrata populations; NE (Northern), WE (Western) and SA (Southern) A. anguilla populations; and ICE (Icelandic population). [file 1471-2148-14-61-S7.pdf]
