# Supplementary material for: Introgressive hybridization and latitudinal admixture clines in North Atlantic eels
Source: BMC Evol Biol. 2014 Mar 28;14:61. doi: 10.1186/1471-2148-14-61 (PMC3986858; doi:10.1186/1471-2148-14-61)
Supplement: Additional file 8 — Discriminatory power of microsatellite markers to detect IBD patterns as depicted by locus-specific FST values. [file 1471-2148-14-61-S8.pdf]

Additional file 8.

Discriminatory power of microsatellite markers to detect IBD patterns as depicted by locus-specific F_{ST} values.

Locus	F_{ST}
Aro121	0.0058
Ang114	0.0092
Aro095	0.0100
Aro063	0.0326
Ang151	0.0183
Ang101	0.0088
Ang075	0.0008
Aro054	0.0410
Aro146	0.1200
